# Different Donor Cell Culture Methods Can Influence the Developmental Ability of Cloned Sheep Embryos

**DOI:** 10.1371/journal.pone.0135344

**Published:** 2015-08-20

**Authors:** LiBing Ma, XiYu Liu, FengMei Wang, XiaoYing He, Shan Chen, WenDa Li

**Affiliations:** 1 School of Mathematics, Physics and Biological Engineering, Inner Mongolia University of Science & Technology, Baotou, Inner Mongolia, China; 2 Baotou Light Industry Vocational Technical College, Baotou, Inner Mongolia, China; USA, UNITED STATES

## Abstract

It was proposed that arresting nuclear donor cells in G0/G1 phase facilitates the development of embryos that are derived from somatic cell nuclear transfer (SCNT). Full confluency or serum starvation is commonly used to arrest *in vitro* cultured somatic cells in G0/G1 phase. However, it is controversial as to whether these two methods have the same efficiency in arresting somatic cells in G0/G1 phase. Moreover, it is unclear whether the cloned embryos have comparable developmental ability after somatic cells are subjected to one of these methods and then used as nuclear donors in SCNT. In the present study, *in vitro* cultured sheep skin fibroblasts were divided into four groups: (1) cultured to 70–80% confluency (control group), (2) cultured to full confluency, (3) starved in low serum medium for 4 d, or (4) cultured to full confluency and then further starved for 4 d. Flow cytometry was used to assay the percentage of fibroblasts in G0/G1 phase, and cell counting was used to assay the viability of the fibroblasts. Then, real-time reverse transcription PCR was used to determine the levels of expression of several cell cycle-related genes. Subsequently, the four groups of fibroblasts were separately used as nuclear donors in SCNT, and the developmental ability and the quality of the cloned embryos were compared. The results showed that the percentage of fibroblasts in G0/G1 phase, the viability of fibroblasts, and the expression levels of cell cycle-related genes was different among the four groups of fibroblasts. Moreover, the quality of the cloned embryos was comparable after these four groups of fibroblasts were separately used as nuclear donors in SCNT. However, cloned embryos derived from fibroblasts that were cultured to full confluency combined with serum starvation had the highest developmental ability. The results of the present study indicate that there are synergistic effects of full confluency and serum starvation on arresting fibroblasts in G0/G1 phase, and the short-term treatment of nuclear donor cells with these two methods could improve the efficiency of SCNT.

## Introduction

In the technology of somatic cell nuclear transfer (SCNT), a differentiated somatic nucleus is transferred into the cytoplasm of a mature, enucleated oocyte; the cytoplasm of the oocyte has the ability to reprogram the somatic nucleus into a totipotent state. Therefore, SCNT-derived embryos of high quality can develop to term. However, the efficiency of SCNT technology is low, and it can be influenced by many factors, such as the quality of the mature oocytes [1], the type, passage number and cell cycle phase of the donor cells [2–4], and the procedure used for SCNT [5].

In early studies, somatic cells arrested in G0/G1 phase were recommended as the ideal nuclear donors in SCNT because they facilitated coordination in the cell cycle of the somatic nuclei and the cytoplasm of oocytes [6, 7]. Moreover, during SCNT, if the nuclear genetic material was totally removed from an oocyte, and a somatic cell in G0/G1 phase was injected (or fused) into this enucleated oocyte, after this reconstructed embryo was activated, and a certain protein synthesis inhibitor was used to prevent the exclusion of genetic material, this SCNT-derived embryo would have the correct number of chromosomes (diploid) [8]. When somatic cells are cultured *in vitro*, the pool of cells exists in different phases of the cell cycle. Therefore, two methods, serum starvation or culture to full confluency, have been used to arrest somatic cells in G0/G1 phase, so that they can be used as nuclear donors in SCNT [7, 9–11]. However, there is some controversy as to whether these two methods can efficiently arrest somatic cells in G0/G1 phase [12–15]. For example, full confluency and serum starvation had the same efficiency in arresting cattle granulosa cells and fibroblasts and canine dermal fibroblasts in G0/G1 phase [12, 13]. However, full confluency was more efficient than serum starvation in arresting goat dermal fibroblasts in G0/G1 phase [15]. In contrast to full confluency, serum starvation for 3 days could more efficiently arrest cat skin fibroblasts in G0/G1 phase [16]. We considered the possibility that somatic cells derived from different species may have a different response to full confluency or serum starvation. Additionally, if somatic cells are subjected to one of these two methods and then used as nuclear donors in SCNT, it is unclear whether the resulting cloned embryos are comparable in developmental ability and quality.

In the present study, *in vitro* cultured sheep skin fibroblasts were differentially cultured to 70–80% confluency (with or without further starvation in low serum medium for 4 d) or full confluency (with or without further starvation in low serum medium for 4 d), and flow cytometry was used to assay the percentage of fibroblasts from each method that was in G0/G1 phase, and cell counting was used to assay the viability of the fibroblasts. Real-time reverse transcription PCR (real-time RT PCR) was used to determine the levels of expression of several cell-cycle-related genes in the differentially cultured fibroblasts. Subsequently, the different groups of fibroblasts were used as nuclear donors, and the developmental ability and the quality of the SCNT-derived embryos were compared.

## Materials and Methods

Unless otherwise indicated, all chemicals were purchased from Sigma-Aldrich Corporation (St. Louis, MO, USA).

### Somatic cells cultured *in vitro* and frozen in liquid nitrogen

Sheep skin fibroblasts were isolated from the ear of a Mongolian sheep (*Ovis aries*) (a healthy, two-year old, virgin ewe) obtained from the Nanqiao slaughterhouse in Baotou city. The primary culture of fibroblasts was performed as previously described with some modifications [17, 18]. Briefly, tissues were mechanically dissociated, and explants were cultured in Dulbecco’s Modified Eagle Medium (DMEM; Gibco, Life Technologies, Carlsbad, CA, USA) supplemented with 10% (v/v) fetal bovine serum (FBS, HyClone, Logan, UT, USA), 100 IU/ml penicillin and 100 μg/ml streptomycin at 38°C in a humidified atmosphere of 5% CO_2_. When the cells from the explants reached 90% confluency, they were removed with 0.25% (m/v) trypsin-0.05% (m/v) ethylenediaminetetraacetic acid (EDTA) treatment, washed 2–3 times in PBS, counted, frozen into aliquots in 10% (v/v) DMSO, 20% (v/v) FBS and 70% (v/v) DMEM, and stored in liquid nitrogen.

### Somatic cell culture methods and cell cycle analysis

Thawed fibroblasts were plated in 12-well plates and cultured in normal DMEM using the conditions described above. When the fibroblasts reached 70–80% confluency (control group), every 1–2 plates of fibroblasts were subjected to one of the following culture methods: low serum starvation (0.5% (v/v) FBS in DMEM) for 4 d or cultured to full confluency in normal DMEM with or without further starvation for 4 d. Three wells of fibroblasts from each treatment (70–80% confluency, full confluency, serum starvation and full confluency with serum starvation) were removed from the plate, washed 2–3 times in PBS, and then immobilized and stained using the Cell Cycle Detection Kit (KeyGen, Nanjing, China) according to the manufacturer’s protocols. Subsequently, the cell cycle of the fibroblasts was assayed with flow cytometry (BD FACSCanto II, Becton, Dickinson and Company, Franklin Lakes, NJ, USA).

All fibroblasts in one well from each culture method were harvested, washed 1–2 times in PBS. After dyed with 0.4% (m/v) trypan blue (dissolved in PBS) for 3 min, the viability ((No. of viable cells/No. of total cells)×100%) of the fibroblasts was assayed by cell counting. For each culture method, three wells of fibroblasts were separately used as three biological replicates for cell counting; and for each biological replicate, three technical replicates were performed, and the mean±standard deviation was calculated.

### Real-time RT PCR

Cell extracts were prepared according to a previously described procedure [19] with slight modifications. For each culture method, three wells of fibroblasts were separately used as three biological replicates for real-time RT PCR; and for each biological replicate, three technical replicates were performed. Briefly, after each culture method, one well of fibroblasts were removed from the plate, washed 2–3 times, and harvested by centrifugation. After the supernatant was discarded, 98 μl of ice-cold Cells-to-cDNA II Cell Lysis Buffer (Ambion, USA) were added to each microcentrifuge tube. The mixture was incubated at 75°C for 15 min, and then placed on ice. After 2 μl of RNase-free DNase I (2 U/μl, Ambion, USA) were added, the mixture was incubated at 37°C for 15 min to degrade the genomic DNA, and the remaining RNA was used as the template for real-time RT PCR. The primers for real-time RT PCR were designed according to the mRNA sequences of *CCNB1* (cyclin B1), *CCNG2* (cyclin G2), *TP53* (tumor protein p53), *TNFRSF17* (tumor necrosis factor receptor superfamily member 17) and *GAPDH* (glyceraldehyde-3-phosphate dehydrogenase) using Primer Premier 5.0 software and are shown in Table 1. Real-time RT PCR was performed using the One Step SYBR PrimeScript PLUS RT-PCR Kit (TaKaRa Biotech. (Dalian) Co., Ltd., Dalian, China) according to the manufacturer’s protocol. The real-time RT PCR mixture consisted of 2 μl total RNA, 1 μl forward primer (10 μM), 1 μl reverse primer (10 μM), 0.5 μl PrimeScript PLUS RTase Mix, 1.5 μl TaKaRa Ex Taq HS Mix, 12.5 μl 2×One Step SYBR RT-PCR Buffer, and 6.5 μl RNase Free dH_2_O in a total volume of 25 μl. Real-time RT PCR was performed by holding the reactions at a specific temperature (45°C for *CCNG2* and *TNFRSF17*; 50°C for *TP53*; 48°C for *CCNB1* and *GAPDH*) for 45 min, a 95°C incubation for 2 min to inactivate the PrimeScript PLUS RTase, and 40 cycles of denaturation at 94°C for 10 s, annealing at a specific temperature (shown in Table 1) for 30 s and extension at 72°C for 30 s using a real-time thermal cycler (Smart Cycler II, Cepheid, Sunnyvale, CA, USA). Next, melt curves were generated by slowly heating (0.2°C/s) the PCR products from 56°C to 95°C. The real-time RT PCR products were further confirmed by electrophoresis. *GAPDH* was used as a reference gene, and the expression of the cell-cycle-related genes (*CCNB1*, *CCNG2*, *TNFRSF17* and *TP53*) from fibroblasts in each group was normalized to that of untreated fibroblasts (control group) using the 2^-ΔΔC^_T_ method.

**Table 1:** The Primers for Real-Time RT PCR.

Gene	GenBank Accession Number	Primer sequences	Annealing temperature	Amplification efficiency	Product length
***CCNB1***	XM_004016916	Sense 5'-AACTAACTATGCTGGACTACGA-3'	52°C	98.8%	146 bp
		Anti-sense 5'-CAGGGATTCTTCAGTGTATG-3'			
***CCNG2***	XM_004009930	Sense 5'-TCGGATTGTTGAACCTCT-3'	48°C	92.0%	134 bp
		Anti-sense 5'-ATCTTCGACTTTGGCATT-3'			
***TP53***	NM_001009403	Sense 5'-CCACCATCCACTACAACTTCA-3'	55°C	101.2%	147 bp
		Anti-sense 5'-CAGGACAGGCACAAACACG-3'			
***TNFRSF17***	XM_004020779	Sense 5'-TAACGCAGACCTGGATGT-3'	48°C	91.8%	214 bp
		Anti-sense 5'-CAGACTGTCGCAGTAGCC-3'			
***GAPDH***	NM_001190390	Sense 5'-ACGTGTCCGTTGTGGATC-3'	52°C	96.7%	158 bp
		Anti-sense 5'-GTGAGTGTCGCTGTTGAAGT-3'			

### Somatic cell nuclear transfer

The procedure for sheep oocyte collection, maturation and the removal of cumulus cells was described previously [18]. Denuded oocytes with a polar body were incubated in H-SOF supplemented with 7.5 μg/ml cytochalasin B, 10 μg/ml Hoechst 33342 and 10% (v/v) FBS at 38.5°C for 15 min, and then mounted onto a micromanipulator (NT-88NE, Nikon-Narishige, Tokyo, Japan) equipped with epifluorescence. The first polar body and adjacent cytoplasm were removed using an aspiration micropipette. Enucleated oocytes were analyzed by exposure to UV light, and only oocytes from which all chromosomes were removed were used for SCNT. One fibroblast was injected into the perivitelline space of an enucleated sheep oocyte using an injection micropipette. The karyoplast-cytoplast couplets were equilibrated in an electrofusion medium (0.3 M mannitol, 0.5 mM HEPES, 1% (m/v) fatty acid-free BSA, 0.05 mM CaCl_2_ and 0.1 mM MgCl_2_) for 3 min, then transferred into a cell fusion chamber containing the same medium for electrofusion using a fusion machine (EP-1 Voltain, CryoLogic Pty. Ltd., Melbourne, Australia). After manual alignment, the karyoplast-cytoplast couplets were subjected to a double DC fusion pulse of 1.25 kV/cm for 80 μs, as described previously [18]. After electrofusion, the karyoplast-cytoplast couplets were transferred into H-SOF supplemented with 10% (v/v) FBS to complete the fusion process. The fused embryos were activated by culturing them in H-SOF containing 5 μM ionomycin and 10% (v/v) FBS for 5 min and were subsequently cultured in SOF containing 2 mM 6-DMAP and 10% (v/v) FBS for 4 h. Activated cloned embryos were washed 2–3 times, and then cultured at 38°C in a humidified atmosphere of 5% CO_2_ in SOF supplemented with 2% (v/v) essential amino acids (Gibco), 1% (v/v) non-essential amino acids (Gibco), 8 mg/ml fatty acid-free BSA, 5% (v/v) FBS and 1 mM glutamine. The progression of embryonic development was monitored every 24 h and half of the culture medium was refreshed every 48 h. All groups of cloned embryos were cultured *in vitro* for 12 days, all blastocysts were counted during culture. The cell number in the early blastocysts (obtained on the 8th day of *in vitro* culture) was assayed to determine the quality of the cloned embryos using a previously described protocol [20]. Briefly, blastocysts were incubated for 30 min at 38°C in PBS supplemented with 1.0 mg/ml Hoechst 33342, then, placed in a drop of mounting medium under a coverslip on a glass slide, the coverslip was slightly pressed, so that all cells in the blastocyst were spread out, and the cell number was counted under an inverted microscope (Ti-U, Nikon, Tokyo, Japan) equipped for epifluorescence. Three blastocysts derived from each group of fibroblasts were assayed.

### Statistical analyses

The number of fibroblasts in the different phases of cell cycle as well as the developmental rate of cloned embryos was compared for statistical significance by the chi-square analysis using SPSS software (SPSS Inc., Chicago, IL, USA). The viability of fibroblasts, the levels of expression for the cell cycle-related genes and the cell number from the cloned early blastocysts were compared for statistical significance by one-way analysis of variance, and further compared statistically by least significant difference (LSD). Differences with a p-value<0.05 were considered to be statistically significant.

## Results

### The cell cycle of differentially cultured fibroblasts

**Fig 1:**
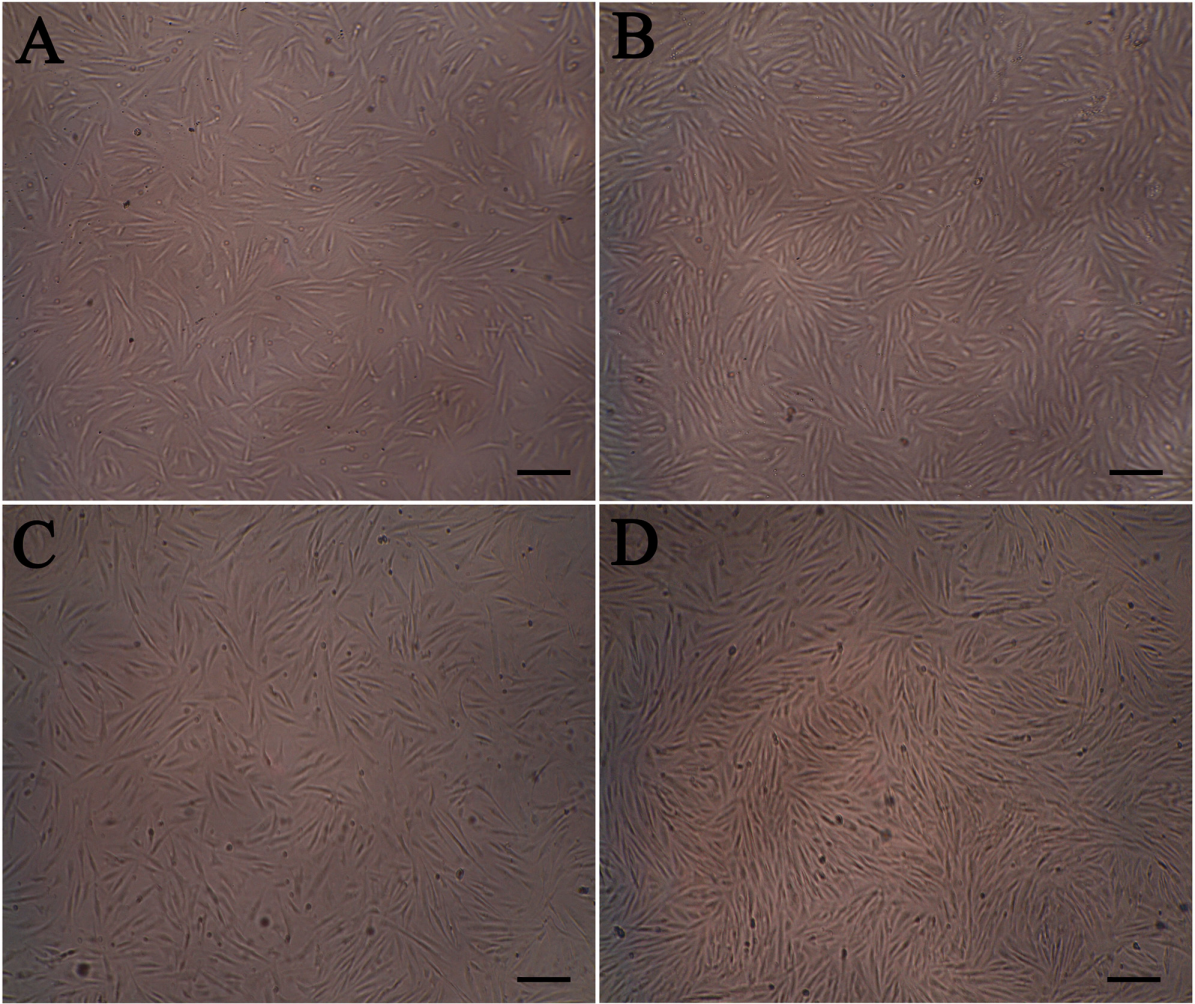
*In Vitro* Cultured Fibroblasts Using Different Methods. A, 70–80% confluency; B, full confluency; C, serum starvation; D, full confluency with serum starvation. Scale bar: 50 μm.

**Fig 2:**
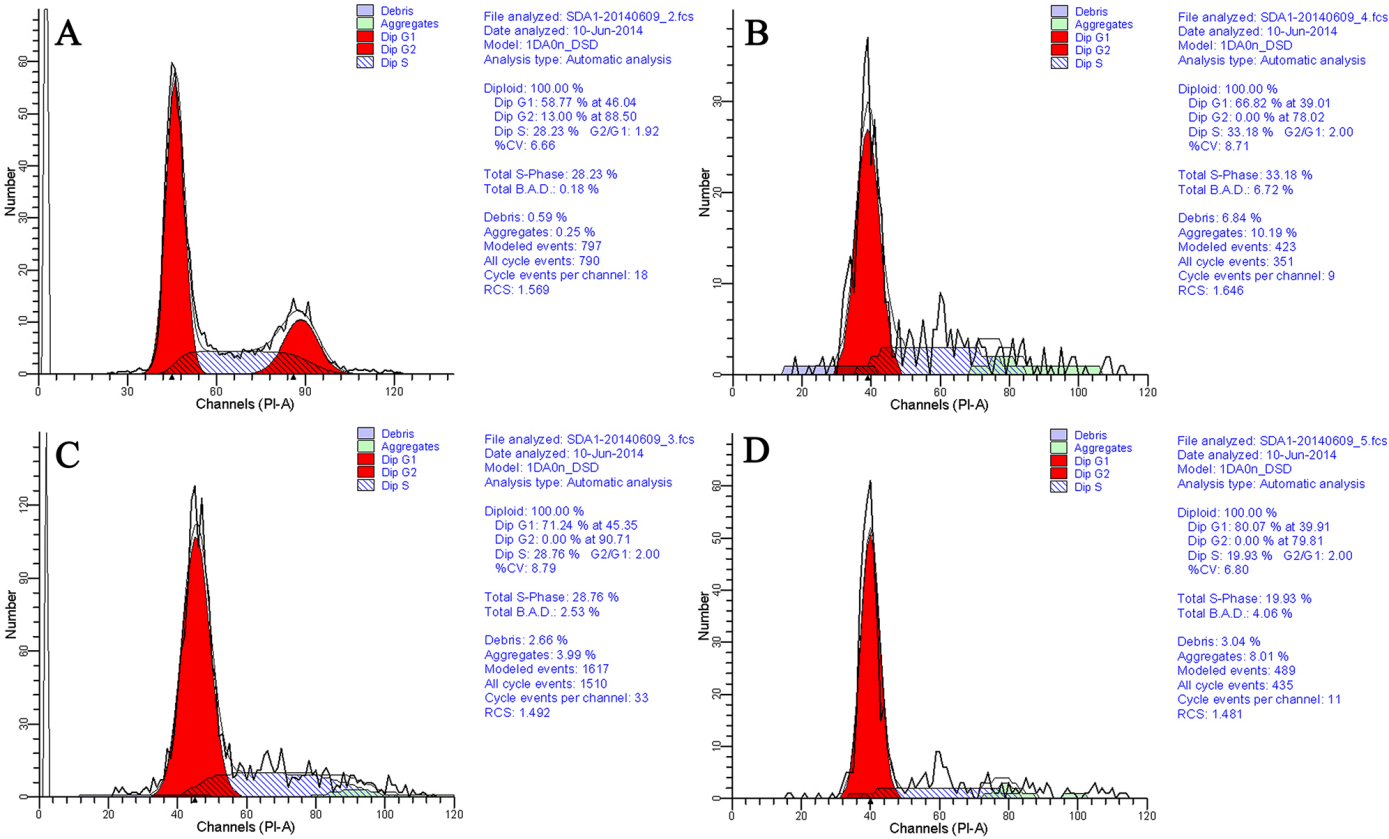
The Percentages of Fibroblasts in Different Phases of the Cell Cycle was Assayed by Flow Cytometry. A, 70–80% confluency; B, full confluency; C, serum starvation; D, full confluency with serum starvation.

After culture with different methods, there was no apparent change in fibroblasts morphology (Fig 1). The percentages of fibroblasts in the different phases of the cell cycle were assayed by flow cytometry, and the results are shown in Fig 2 and Table 2.

**Table 2:** The Percentages of Fibroblasts in Different Phases of the Cell Cycle under Different Culture Methods.

Treatments	The percentages of fibroblasts in different phases of the cell cycle*		
	G0/G1 phase	S phase	G2/M phase
**Control** **	58.77^a^	28.23^a^	13.00
**Full confluency**	66.82^b^	33.18^a^	0.00
**Serum starvation**	71.24^b^	28.76^a^	0.00
**Full confluency+serum starvation**	80.07^c^	19.93^b^	0.00

^a, b, c^ Values with different superscripts within the same column are significantly different (P<0.05).

*Data were collected from one series of flow cytometry assay, and significant differences were determined by comparing the number of fibroblasts in the different phases of the cell cycle.

** Control: fibroblasts were cultured to 70–80% confluency.

As shown in Table 2, the effect of full confluency or serum starvation on the cell cycle of fibroblasts was similar; both culture methods significantly (p<0.05) increased the percentage of fibroblasts in G0/G1 phase and decreased the percentages of fibroblasts in S and G2/M phases. Of the three culture methods, full confluency combined with serum starvation was the most efficient in arresting fibroblasts in G0/G1 phase, as the percentage of fibroblasts in G0/G1 phase was the highest in this group. In contrast, the percentage of fibroblasts in S phase was lowest in this group. Moreover, all of the culture methods could efficiently inhibit fibroblasts from entering into G2/M phase because none of the fibroblasts remained in G2/M phase in any of the groups.

The viability of fibroblasts in the groups of 70–80% confluency, full confluency, serum starvation and full confluency with serum starvation were 92.47±3.07%, 90.30±2.66%, 78.87±3.40% and 76.30±3.09%, respectively. The difference in cell viability between 70–80% confluency group and full confluency group was not significant, the same result was also obtained between serum starvation group and full confluency with serum starvation group. However, the latter two groups had significantly (p<0.05) lower cell viability than those of the former two groups.

### Expression of cell cycle-related genes in differentially cultured fibroblasts

**Fig 3:**
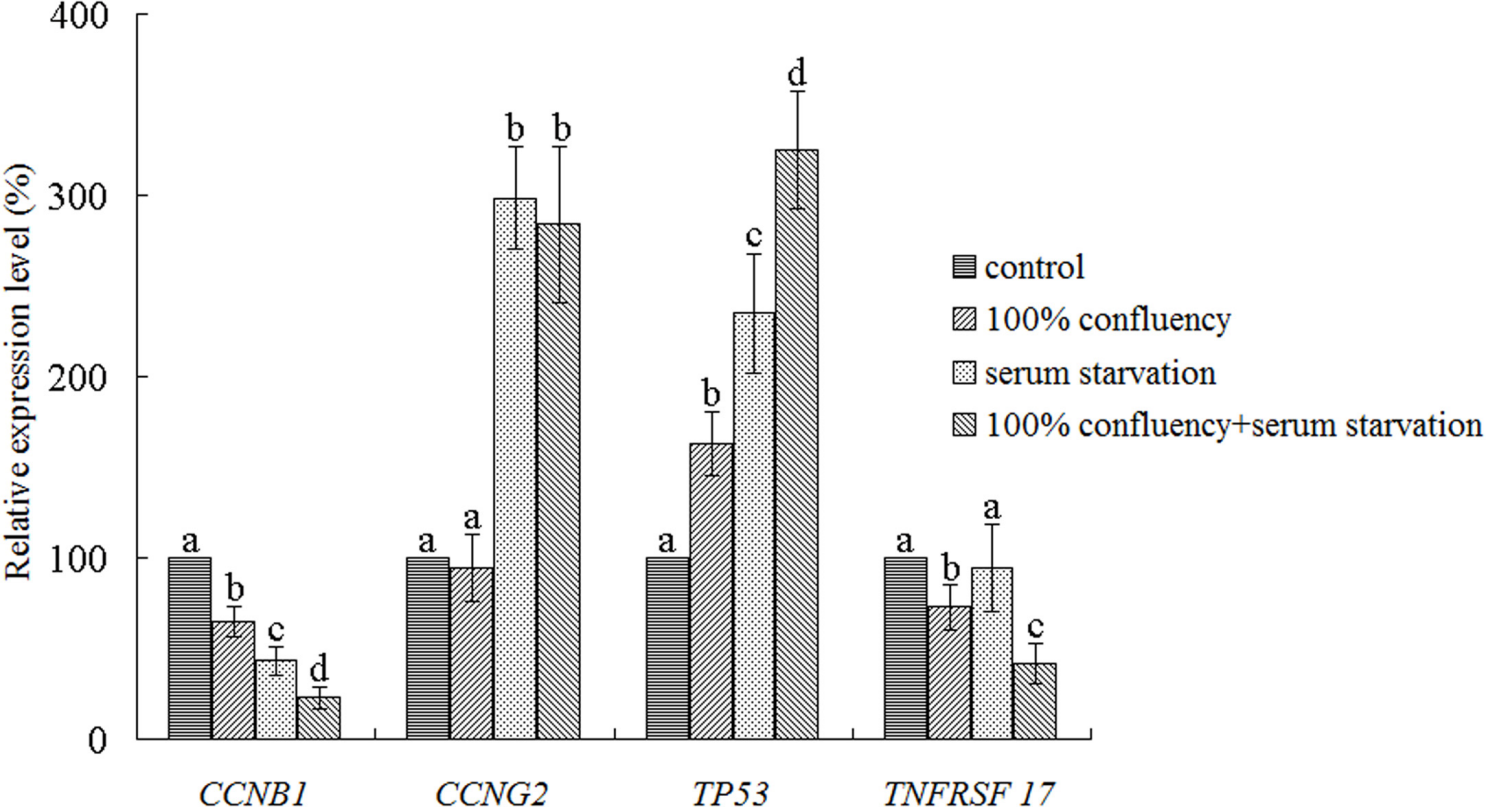
The Relative Expression of Cell Cycle-Related Genes was Assayed by Real-Time RT PCR. The expression of genes in each group was normalized to the control group. Data were collected from three replicates and are presented as the mean±standard deviation. Bars with different lowercase letters are significantly different (p<0.05).

Real-time RT PCR was used to assay the effects of the different culture methods on the expression levels of cell cycle-related genes, and the results are shown in Fig 3.

After differential culture methods, the expression of cell cycle-related genes was different. The expression of *CCNB1*, a gene related to cell cycle and division, was decreased in all groups. Compared to the control group, the levels of *CCNB1* expression were 64.6±8.4%, 43.3±7.9% and 22.8±6.0% in the full confluency, serum starvation and full confluency with serum starvation groups, respectively.

*CCNG2* expression, another gene related to cell cycle and division, was increased in the serum starvation and full confluency with serum starvation groups. Compared to the control group, the levels of *CCNG2* expression were 298.6±28.1% and 283.9±43.4% in these two groups, respectively. However, in the full confluency group, *CCNG2* expression was comparable to the control group (94.6±18.5% relative to control group).

*TP53* is a tumor suppressor gene, and the protein encoded by this gene can arrest a cell at the G1/S regulation point to inhibit cell proliferation. Compared to the control group, *TP53* expression increased to 162.9±17.4%, 234.7±33.3% and 324.7±32.4% in the full confluency, serum starvation and full confluency with serum starvation groups, respectively.

The protein encoded by *TNFRSF17* (also designated as B-cell maturation antigen (BCMA)), a member of the TNF-receptor superfamily, is important for B cell development and autoimmune response. Moreover, this protein can bind to various members of the TNFR-associated factor family, such as APRIL, to promote cell growth and proliferation [21, 22]. The effects of the different culture methods on *TNFRSF17* expression were different. Both full confluency and full confluency with serum starvation could decrease *TNFRSF17* expression to 72.9±12.7% and 41.4±11.1% relative to the control group. However, after serum starvation, *TNFRSF17* expression remained comparable to the control group (94.4±24.5%).

### The development and quality of cloned embryos derived from differentially cultured fibroblasts

**Fig 4:**
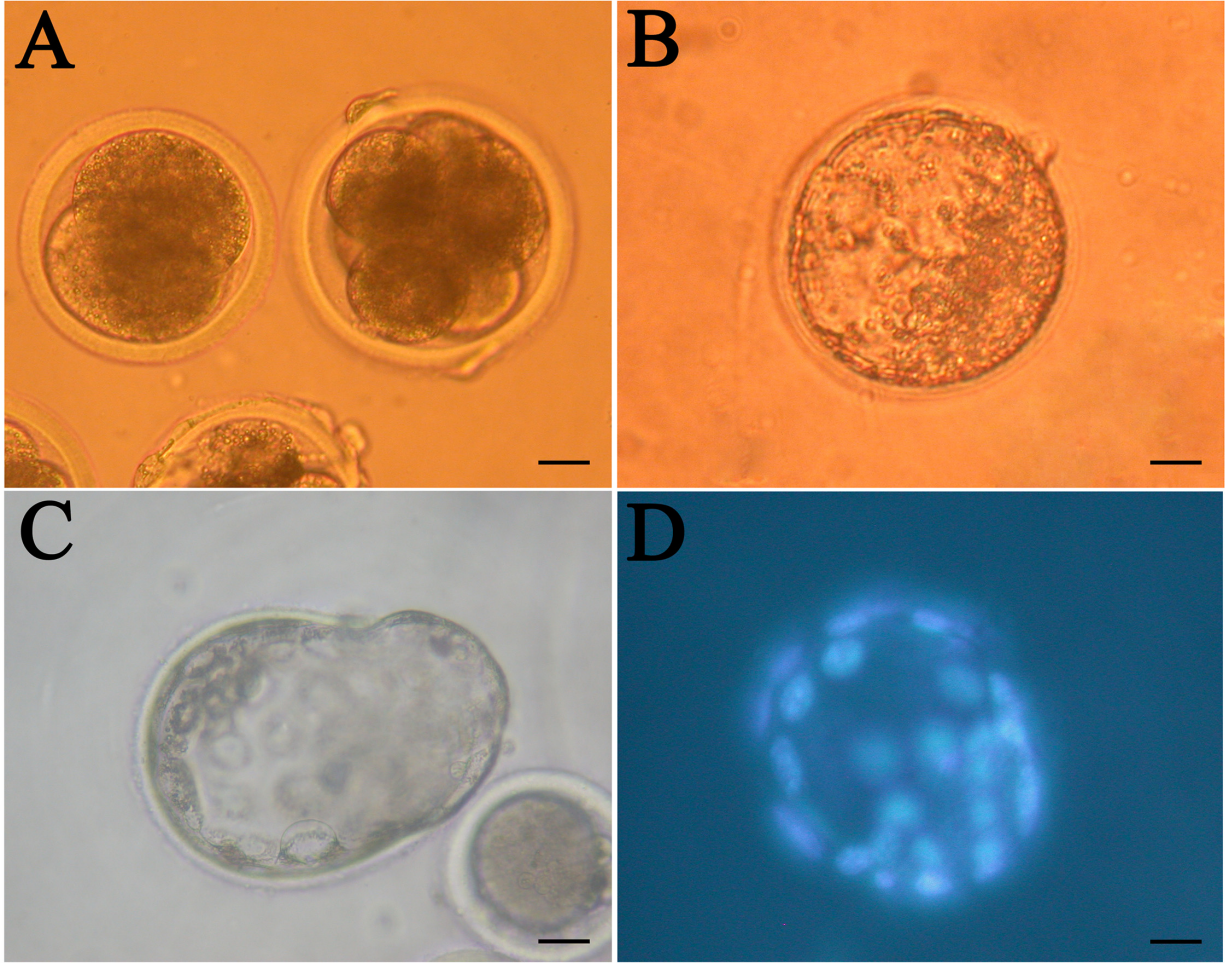
*In Vitro* Cultured Cloned Sheep Embryos. A, 2- and 8-cell embryos; B, early blastocyst; C, hatching blastocyst; D, early blastocyst stained with Hoechst 33342. Scale bar: 20 μm.

After culture using different methods, fibroblasts were used as nuclear donors for SCNT. The cloned embryos could develop to blastocysts in 7–8 days and the blastocysts hatched in 9–10 days (Fig 4). The developmental ability of the cloned embryos in the present study was comparable with the results of our and other previous studies [18, 23, 24], and the results are shown in Table 3.

**Table 3:** The Developmental Ability of Cloned Embryos Derived from Differentially Cultured Fibroblasts.

Treatments	No. cultured embryos	2-cell	4-cell	8-cell	morula	blastocyst**
**Control** *	145	65 (44.8%)^a^	48 (33.1%)^a^	37 (25.5%)^a^	28 (19.3%)^a^	9 (6.2%)^a^
**Full confluency**	132	68 (51.5%)^a^	55 (41.7%)^a^ ^b^	46 (34.8%)^a^ ^b^	36 (27.3%)^a^ ^b^	13 (9.8%)^a^ ^b^
**Serum starvation**	101	57 (56.4%)^a^ ^b^	47 (46.5%)^b^	41 (40.6%)^b^	34 (33.7%)^b^	12 (11.9%)^a^ ^b^
**Full confluency+serum starvation**	127	82 (64.6%)^b^	68 (53.5%)^b^	58 (45.7%)^b^	49 (38.6%)^b^	22 (17.3%)^b^

^a, b^Values with different superscripts within the same column are significantly different (P<0.05).

*Control: fibroblasts were cultured to 70–80% confluency.

**The data include all blastocysts from 12 days of culture.

Data were collected from 10 series of SCNT experiments.

Compared with embryos derived from fibroblasts in the control group, embryos derived from fibroblasts cultured in serum starvation or full confluency with serum starvation conditions had a significantly (p<0.05) higher rates of 4-cell, 8-cell and morula stages. However, the use of full confluency fibroblasts only slightly increased the developmental ability of cloned embryos. In all developmental stages, the difference in developmental rate of the cloned embryos was not significant (p>0.05) between the control group and the full confluency group. Moreover, of the three culture methods, full confluency with serum starvation showed the greatest increase in the developmental ability of the cloned embryos.

The cell number in cloned early blastocysts (obtained on the 8th day of *in vitro* culture) was assayed after staining with Hoechst 33342 (Fig 4). There was no significant difference in the cell number of cloned blastocysts derived from 70–80% confluency (105.7±7.0), full confluency (96.3±8.7), serum starvation (95.7±6.5) and full confluency with serum starvation (101.7±16.8), respectively. This result suggests that the different fibroblasts culture methods did not affect the quality of the resulting cloned embryos.

## Discussion

Presently, two types of methods, full confluency (or contact inhibition) and serum starvation, are commonly used to arrest *in vitro* cultured somatic cells in G0/G1 phase. More than half a century ago, it was discovered that non-malignant cells would stop proliferating when they reached full confluence, despite the availability of extracellular nutrients and growth factors [25]. Recently, it was proposed that full confluency could upregulate p27^Kip1^ through the p38α-Spry2-EGFR pathway, to arrest cell proliferation [26]. Serum starvation can also lead to cell cycle arrest in G0/G1 phase [27]. However, its mechanism may be different from that of full confluency. It was proposed that serum starvation arrested the cell cycle through the Skp2-p27-CDK2 (or CDK4) pathway [28]. In the present study, both the percentage of fibroblasts in G0/G1 phase and the expression levels of cell cycle-related genes were different after three types of culture methods. These results further implied that the mechanisms of arresting cell cycle by the previously described methods were different. For example, full confluency could result in a significant decrease in *TNFRSF17* expression, but serum starvation had little effect on the expression of this gene. Similar results were also obtained in other studies [29–31]. The biological relevance of BCMA, the protein encoded by *TNFRSF17*, in maintaining the viability and proliferation of Hodgkin and Reed-Sternberg (HRS) lymphoma cells has been demonstrated by Chiu et al [32]. APRIL and BAFF could deliver nonredundant signals via BCMA and TACI receptors through both autocrine and paracrine pathways. These signals caused NF-κB activation; antiapoptotic BCL-2 and BCL-X_L_ up-regulation, proapoptotic BAX down-regulation, growth-inducing c-MYC protein up-regulation, and as a result, the survival and proliferation of HRS cells were enhanced [32]. In fact, cell cycle arrest upon confluency may be evoked by cell-cell communication, which is transduced into the cytoplasm and nucleus through certain signaling pathways [33, 34]. When cells were cultured in low serum medium, they could not obtain sufficient growth factors for their proliferation. Subsequently, growth factor-related signaling pathways and metabolic pathways were affected, resulting in cell cycle arrest [16, 35].

There was a controversy as to whether full confluency and serum starvation had the same efficiency in arresting *in vitro* cultured cells in G0/G1 phase because similar and different efficiencies have been reported [12–15]. We considered that this contradiction may be because cells derived from different species had distinct responses to different culture methods, and the time of culture was also a pivotal factor in arrest efficiency. For example, different methods (full confluency, serum starvation and chemical inhibitors) had different levels of efficiency in G0/G1 phase arrest in fibroblasts derived from different species of cats [36]. In addition, prolonging the time of serum starvation could more efficiently arrest goat fibroblasts in G0/G1 phase [15]. In the present study, full confluency and serum starvation had the same efficiency in arresting sheep skin fibroblasts in G0/G1 phase. Moreover, the synergistic effects of these two types of methods could further increase the percentage of fibroblasts in G0/G1 phase. Similar results were also obtained in another study using domestic cat fibroblasts [14]. As discussed above, although prolonging the time of serum starvation could improve arrest efficiency, the percentage of dead cells also increased [15]. Therefore, short-term culture with a combination of these two methods may be an alternative strategy.

In present study, the blastocyst rate of cloned embryos in control group was as low as 6.2% (No. of blastocysts/No. of cultured embryos). This result was comparable with the result of a recent sheep SCNT study, in which fresh lymphocytes were used as nuclear donors, and sheep enucleated MII oocytes were used as nuclear recipients. The blastocyst rate of cloned embryos was also as low as 7.0% (25/356, No. of blastocysts/No. of cultured embryos) [23]. Therefore, it was considered that arresting donor cells in G0/G1 phase facilitated the development of cloned embryos derived from SCNT [8]. In previous studies of SCNT, serum starvation or full confluency was commonly used to culture nuclear donors [7, 9–11], and the combination of these two methods for culturing nuclear donors was only reported in few studies [18, 19]. In a bovine SCNT study, nuclear donors (bovine fibroblasts) were cultured with serum starvation or to full confluency, and the cloned embryos had a similar ability to develop into blastocysts [12]. The results of the present study were in accord with this study; the blastocyst rates of cloned embryos in the groups of full confluency and serum starvation were 9.8% and 11.9% (No. of blastocysts/No. of cultured embryos), respectively. Our results were comparable with the results of a recent sheep SCNT study, in which sheep confluent cumulus cells were used as nuclear donors, and sheep enucleated MII oocytes were used as nuclear recipients; the blastocyst rates of cloned embryos were 7.1%-11.7% (5/70-11/94, No. of blastocysts/No. of cultured embryos) [24]. In fact, in our a recent study, sheep skin fibroblasts were cultured to full confluency with further serum starvation for 3–5 d and then used as nuclear donors, sheep enucleated MII oocytes were used as nuclear recipients, the blastocyst rate of cloned embryos was 14.6% (15/103, No. of blastocysts/No. of cultured embryos) [18], this result was comparable with the result of present study (17.3%, No. of blastocysts/No. of cultured embryos). However, in that study, we did not further study the effects of different donor cell culture methods on the developmental ability of sheep cloned embryos.

In the present study, full confluency or serum starvation could efficiently arrest fibroblasts in G0/G1 phase. However, cloned embryos derived from these fibroblasts had a comparable total developmental ability with those derived from untreated fibroblasts, and only fibroblasts cultured to full confluency with serum starvation could produce cloned embryos with a significantly higher developmental ability. As discussed above, the mechanism of arresting cell cycle by full confluency or serum starvation was different. A combination of these two methods could arrest somatic cells in a more stable G0/G1 phase. After these pretreated somatic cells were transferred into the cytoplasm of enucleated oocytes, the nuclei and cytoplasm of the cloned embryos were coordinated in the cell cycle, which facilitated the development of cloned embryos. Moreover, many genes were downregulated in cells arrested in G0/G1 phase [31], which may have facilitated the reprogramming of the somatic nuclei and the development of cloned embryos. Therefore, we propose that full confluency combined with serum starvation could more efficiently arrest *in vitro* cultured somatic cells in G0/G1 phase, and when these cells were used as nuclear donors, the resulting cloned embryos would exhibit higher developmental ability. This finding may improve the efficiency of SCNT.
